# Identifying longitudinal cognitive resilience from cross-sectional amyloid, tau, and neurodegeneration

**DOI:** 10.1186/s13195-024-01510-y

**Published:** 2024-07-03

**Authors:** Rory Boyle, Diana L. Townsend, Hannah M. Klinger, Catherine E. Scanlon, Ziwen Yuan, Gillian T. Coughlan, Mabel Seto, Zahra Shirzadi, Wai-Ying Wendy Yau, Roos J. Jutten, Christoph Schneider, Michelle E. Farrell, Bernard J. Hanseeuw, Elizabeth C. Mormino, Hyun-Sik Yang, Kathryn V. Papp, Rebecca E. Amariglio, Heidi I. L. Jacobs, Julie C. Price, Jasmeer P. Chhatwal, Aaron P. Schultz, Michael J. Properzi, Dorene M. Rentz, Keith A. Johnson, Reisa A. Sperling, Timothy J. Hohman, Michael C. Donohue, Rachel F. Buckley, for the Alzheimer’s Disease Neuroimaging Initiative

**Affiliations:** 1 Department of Neurology, Massachusetts General Hospital, Harvard Medical School, Boston, MA USA; 2 Department of Neurology, Brigham and Women’s Hospital, Harvard Medical School, Boston, MA USA; 3 Department of Radiology, Massachusetts General Hospital, Harvard Medical School, Boston, MA USA; 4 Department of Neurology, Institute of Neuroscience, Cliniques Universitaires SaintLuc, Université Catholique de Louvain, Brussels, Belgium; 5 Department of Neurology and Neurological Sciences, Stanford University School of Medicine, Stanford, CA USA; 6 Wu Tsai Neuroscience Institute, Stanford, CA USA; 7 Faculty of Health, Medicine and Life Sciences, School for Mental Health and Neuroscience, Alzheimer Centre Limburg, Maastricht University, Maastricht, The Netherlands; 8 Department of Neurology, Vanderbilt University Medical Center, Nashville, TN USA; 9 Alzheimer’s Therapeutic Research Institute, University of Southern California, San Diego, CA USA; 10 Melbourne School of Psychological Sciences, University of Melbourne, Melbourne, VIC Australia

**Keywords:** Longitudinal analysis, Alzheimer’s disease, Amyloid, Tau, PET, MRI, Cognition, Cognitive Reserve, Cognitive Resilience

## Abstract

**Background:**

Leveraging Alzheimer’s disease (AD) imaging biomarkers and longitudinal cognitive data may allow us to establish evidence of cognitive resilience (CR) to AD pathology *in-vivo*. Here, we applied latent class mixture modeling, adjusting for sex, baseline age, and neuroimaging biomarkers of amyloid, tau and neurodegeneration, to a sample of cognitively unimpaired older adults to identify longitudinal trajectories of CR.

**Methods:**

We identified 200 Harvard Aging Brain Study (HABS) participants (mean age = 71.89 years, SD = 9.41 years, 59% women) who were cognitively unimpaired at baseline with 2 or more timepoints of cognitive assessment following a single amyloid-PET, tau-PET and structural MRI. We examined latent class mixture models with longitudinal cognition as the dependent variable and time from baseline, baseline age, sex, neocortical Aβ, entorhinal tau, and adjusted hippocampal volume as independent variables. We then examined group differences in CR-related factors across the identified subgroups from a favored model. Finally, we applied our favored model to a dataset from the Alzheimer’s Disease Neuroimaging Initiative (ADNI; n = 160, mean age = 73.9 years, SD = 7.6 years, 60% women).

**Results:**

The favored model identified 3 latent subgroups, which we labelled as Normal (71% of HABS sample), Resilient (22.5%) and Declining (6.5%) subgroups. The Resilient subgroup exhibited higher baseline cognitive performance and a stable cognitive slope. They were differentiated from other groups by higher levels of verbal intelligence and past cognitive activity. In ADNI, this model identified a larger Normal subgroup (88.1%), a smaller Resilient subgroup (6.3%) and a Declining group (5.6%) with a lower cognitive baseline.

**Conclusion:**

These findings demonstrate the value of data-driven approaches to identify longitudinal CR groups in preclinical AD. With such an approach, we identified a CR subgroup who reflected expected characteristics based on previous literature, higher levels of verbal intelligence and past cognitive activity.

**Supplementary Information:**

The online version contains supplementary material available at 10.1186/s13195-024-01510-y.

## Background

Cognitive resilience (CR) refers to a property of the brain that enables better-than-expected cognitive performance given age-related brain change, injury or disease [1, 2]. In Alzheimer’s disease, individuals with higher CR show a reduced risk and/or delayed onset of dementia [3]**.** CR is thought to be a dynamic construct, that is influenced by genetics, lifestyle and environmental factors, and that can change over the life course [4]. Identifying and characterizing longitudinal CR can help us to better understand the factors that predict an individual’s likelihood of performing well in the face of pathological insult. To-date, however, the clinical potential of CR has yet to be fully realized and has been limited to the use of CR-related factors, education [5] and verbal intelligence [6] to adjust normative cognitive data for detecting cognitive impairment.

Difficulties in measuring the latent construct of CR *in-vivo* are a considerable barrier to achieving translational impact of CR research. Post-mortem studies suggest that CR is relatively common, demonstrated in 20–39% of post-mortem cases that possess pathologic AD diagnoses without cognitive impairment at death [7–9]. Identifying CR *in-vivo* is more difficult as, unlike in post-mortem studies, cognitive performance may continue to evolve. Instead, CR has been studied *in-vivo*, and at a cross-section, using sociobehavioral proxy variables (e.g. educational attainment) that reflect the degree of exposure to CR-related life experiences. Another popular approach is to use residuals, obtained from regressing cognitive performance on demographics (e.g. age, sex) and brain structure or pathology (e.g. adjusted hippocampal volume, neocortical Aβ). Despite their widespread use, proxies do not consistently show CR effects [10, 11] and may be associated with cognitive decline via pathways other than CR [12]. For example, educational attainment is associated with socioeconomic status [13] which is associated with cognitive decline via various other pathways such as access to healthcare, the neighborhood environment, and exposure to air pollution [14–16]. CR residuals necessarily contain substantial measurement error [17] and their association with variables of interest may be driven by baseline cognitive levels rather than CR [18]. Along with the absence of a gold-standard endpoint, these issues compound the difficulty of identifying CR in-vivo.

The latent class mixture model (LCMM) [19] is a data-driven modelling technique that has been applied in epidemiological research to identify distinct subgroups of body mass index trajectories [20–22] and of treatment response trajectories in rheumatoid arthritis [23]. As these identified subgroups differ due to a latent variable or process, this method provides a conceptually well-suited approach to investigating CR, which is a latent construct [24]. This approach to studying longitudinal CR allows us to harness as much within-subject information as possible. This can be particularly helpful in the context of early or preclinical stages of disease where cross-sectional differences in cognitive performance may be minimal.

While similar analytic approaches have been applied in AD using post-mortem cohorts [25–27], there has only been one single application – to our knowledge – of the LCMM in prospective cohorts in AD, where three distinct subgroups of cognitive decline (slow decliners, rapid decliners, and severely-impaired decliners) were identified in 1,160 AD patients [28]. Biomarkers of AD pathology and neurodegeneration were not considered in that study and therefore differences in cognitive decline across the identified subgroups may have arisen due to differences in levels of these biomarkers. By adjusting for demographics as well as biomarkers of AD pathology and neurodegeneration, we may be able to identify subgroups that differ due to some other latent factor, i.e. CR.

Here, we applied latent class mixture modeling to identify latent cognitive change subgroups in a sample of clinically unimpaired individuals from the Harvard Aging Brain Study, controlling for sex, baseline age, and biomarkers of AD pathology and neurodegeneration. We compared these latent subgroups based on many CR proxies (including educational attainment, past and current cognitive activity levels, verbal intelligence, occupational complexity, neighborhood disadvantage). Finally, we then applied our model to an Alzheimer’s Disease Neuroimaging Initiative (ADNI) dataset.

## Methods

### Participants

Our first cohort was selected from the Harvard Aging Brain Study (HABS) participants. At study entry, all participants had a global Clinical Dementia Rating of 0, education-adjusted Mini-Mental State Examination score of 27 or greater, a modified Hachinski ischemic score less than or equal to 4 and did not have history of stroke or evidence of infarcts with persistent neurological deficits. Data were collected from April 2010 through April 2023. The HABS protocol and procedures were approved by The Mass General Brigham Institutional Review Board and all participants signed a written informed consent prior to the completion of any study procedures. The study was carried out in accordance with the guidelines of the Declaration of Helsinki.

We identified 200 participants from the HABS dataset (see Table 1) with the following criteria: first cognitive assessment within 1 year of the participant’s first ^18^F-Flortaucipir(FTP)-PET scan (median weeks between cognitive baseline and tau-PET = 17, min = 0, max = 52, see eFig. 1), cognitively unimpaired at analysis baseline with at least 2 timepoints of neuropsychological assessment (median 6 cognitive timepoints, range = 2–10 timepoints, median 1.05 years between assessments), and had available data from an ^11^C-Pittsburgh compound-B(PiB)-PET scan (median weeks between cognitive baseline and PiB-PET = 13, min = 0, max = 116) and an MRI (median weeks between cognitive baseline and MRI = 10, min = 0, max = 62). FTP-PET was introduced into HABS mid-study, with these participants undergoing their first FTP-PET at 2.4 ± 1.9 years after the baseline visit. As such, we did not include retrospective cognitive time points given that we were unclear of their prior level of tau pathological burden. We also ensured that data for CR-related factors were obtained within 1 year of their first FTP-PET scan. The only exception were those data collected from HABS study baseline: educational attainment, verbal intelligence, occupational complexity, past cognitive activity, neighborhood disadvantage, and cardiovascular disease risk scores (available only at HABS study baseline).

**Table 1:** Baseline characteristics of HABS and ADNI datasets

**Characteristic**	**HABS** *N* = 200^1^	**ADNI** *N* = 160^1^	***p*-value**^2^
**Age**	71.89 (9.41)	73.90 (7.60)	0.072
**Sex**			0.8
F	118 (59%)	96 (60%)	
M	82 (41%)	64 (40%)	
**Years of Education**	16.28 (2.86)	16.63 (2.41)	0.4
**Race**			0.4
White	164 (84%)	140 (89%)	
Black or African American	29 (15%)	16 (10%)	
American Indian or Native Alaskan	2 (1.0%)	1 (0.6%)	
American Indian or Native American/White	1 (0.5%)	0 (0%)	
Asian	0 (0%)	1 (0.6%)	
Unknown	4	2	
**Ethnicity**			0.016
Not Hispanic or Latino	197 (99%)	149 (94%)	
Hispanic or Latino	3 (1.5%)	10 (6.3%)	
Unknown	0	1	
**APOE e4 Status**			0.019
e4-	143 (72%)	91 (60%)	
e4 +	55 (28%)	60 (40%)	
Missing	2	9	
**PACC-5**	0.24 (0.71)	0.05 (0.55)	0.003
**Follow up duration**	5.91 (2.13)	3.19 (1.19)	< 0.001
**Number of cognitive timepoints**	5.94 (1.85)	2.78 (0.74)	< 0.001
**Entorhinal Tau Burden**	1.33 (0.28)	1.71 (0.29)	< 0.001
Neocortical Amyloid Burden^*^	1.17 (0.19)	1.14 (0.20)	^*^
**Adj. Hippocampal Volume**	7,492.70 (834.46)	7,427.77 (822.20)	0.5

^1^Mean (SD); n (%). ^2^Wilcoxon rank sum test; Pearson's Chi-squared test

^*^Neocortical amyloid burden not compared across amyloid DVR values in HABS and amyloid SUVR values in ADNI

We identified 160 participants from the ADNI dataset (see Table 1) who had at least 2 timepoints of neuropsychological assessment (median 3 cognitive timepoints, range = 2–5 timepoints, median 1.92 years between assessments) using the same selection criteria as in HABS (median weeks between cognitive baseline and tau-PET = 1 [min = 0, max = 50], PiB-PET = 101 [min = 0, max = 157], MRI = 3 [min = 0, max = 60], see eFig. 1). The ADNI was launched in 2003 as a public–private partnership, led by Principal Investigator Michael W. Weiner, MD. The primary goal of ADNI has been to test whether serial magnetic resonance imaging (MRI), positron emission tomography (PET), other biological markers, and clinical and neuropsychological assessment can be combined to measure the progression of mild cognitive impairment (MCI) and early Alzheimer’s disease (AD). For up-to-date information, see www.adni-info.org. The study was carried out in accordance with the guidelines of the Declaration of Helsinki.

The HABS and ADNI datasets differed on length of available cognitive follow up (see Table 1), baseline cognitive, Aβ and tau levels, duration between baseline biomarker measurements (i.e. neuroimaging scans) and duration between baseline cognitive assessment and baseline biomarker measurements (see eFig. 1).

### Hippocampal volume

Magnetic resonance imaging was performed at MGH on a 3T scanner (TIM Trio; Siemens) with a 12-channel phased-array head coil. A T1-weighted volumetric magnetization–prepared rapid-acquisition gradient-echo (MPRAGE) image was acquired with following parameters: TR = 2300 ms, TE = 2.95 ms, TI = 900 ms, flip angle = 9°, resolution = 1.1 × 1.1 × 1.2 mm. T1-weighted images were processed and quality-assessed using the automated reconstruction protocol in FreeSurfer (version 6.0). Left and right hippocampal volumes were summed and then bilateral hippocampal volumes were corrected for estimated intracranial volume (eTIV) using the regression method with the following Eq [29].:

$$\text{adjusted hippocampal volume }=\text{ raw hippocampal volume}- b(\text{eTIV}-\text{ mean eTIV})$$

$$b=\text{regression coefficient from regression of raw hippocampal volume on eTIV}$$

Fully processed hippocampal volume and eTIV values were obtained for ADNI participants from LONI. We excluded scans which failed UCSF quality control standards for processing and segmentation of the overall scan. Adjusted hippocampal volume was calculated following the aforementioned method.

### Aβ-PET imaging

For HABS, a summary measure of neocortical Aβ was measured using images acquired with a 60-min dynamic acquisition following injection of PiB-PET radiotracer. Imaging was performed on the ECAT EXACT HR^+^ scanner (Siemens) using a previously described protocol [29]. Images were co-registered to their T1-weighted image using Freesurfer-based (version 6.0) regions of interest and then mapped into native PET space using SPM12. A composite measure of neocortical Aβ was calculated as a distribution volume ratio (DVR) across frontal, lateral temporal, parietal and retrosplenial (FLR) regions with the cerebellar gray as the reference region.

Fully processed neocortical Aβ values were obtained for ADNI participants from LONI, where processing and quality control methods are fully described [30]. An FLR-comparable standardized uptake value ratio (SUVR) composite (based on the 50–70 min time window post-injection) was obtained using a ^18^F-florbetapir tracer and was referenced to the whole cerebellum.

### Tau-PET imaging

Tau pathology was measured using images acquired over either 80–100 min or 75–105 min following injection of FTP-PET tracer. In HABS, imaging was performed on the ECAT HR^+^ scanner (Siemens) using a previously described protocol [31]. Images were co-registered to their T1-weighted image using Freesurfer-based (version 6.0) regions of interest and then mapped into native PET space using SPM12. Tau-PET data were computed as SUVRs, with cerebellar gray matter as the reference region in HABS, and we obtained SUVRs for the entorhinal and inferior temporal lobe regions. These data were partial volume corrected using the geometric transfer matrix method [32].

For ADNI, processed SUVR values, partial volume corrected using the geometric transfer matrix method [32], were obtained from LONI. Processing and quality control methods have been fully described elsewhere [33]. As the favored LCMM in HABS was identified using entorhinal tau SUVR (see below in Results), we sought to apply a model using the same covariates in ADNI. As such, for ADNI we obtained the entorhinal tau SUVR value only, which was referenced to the inferior cerebellum.

### Cognitive performance

The Preclinical Alzheimer’s Cognitive Composite-5 (PACC-5) was used to assess cognition. This composite measure is sensitive to Aβ-related cognitive decline in preclinical AD [34, 35] and is calculated as the average of z-scores, based on the mean and standard deviation of the HABS cohort at baseline. It consists of five neuropsychological tests: Mini Mental State Examination, Logical Memory Delayed Recall, Digit-Symbol Substitution Test, Free and Cued Selective Reminding Test (both cued and free recall), and Category Fluency.

An equivalent version of the PACC-5 was calculated in ADNI using the Mini-Mental State Examination, Logical Memory Delayed Recall, Trail Making Test Part B – Time to Complete, Alzheimer’s Disease Assessment Scale-Cognitive Subscale Delayed Word Recall, and Category Fluency – Animals [34]. Each measure was z-scored using study baseline data from cognitively unimpaired individuals across the wider ADNI cohort. Despite differences in the constituent measures of the PACC-5 in both cohorts, the PACC-5 displays relative consistency of the baseline and slopes in HABS and ADNI [36].

### Cognitive resilience-related factors

Several factors that have been associated with CR were measured in HABS.

**Educational attainment** was measured by years of formal education.

**Verbal intelligence** was measured using estimated verbal IQ from the American National Adult Reading test (AMNART VIQ) [37, 38].

**Occupational complexity** was assessed using a summary ‘Data People Things’ score [39] obtained using numerical ratings of each job title from the Dictionary of Occupational Titles (DOT: www.occupationalinfo.org). This score assesses the complexity of an occupation with regards to working with data, with people, and with things. Participants provided the job title for their ‘highest level’ lifetime occupation. Ratings for each dimension were reversed (such that higher scores reflected greater complexity) and then summed to create a total occupational complexity score [10], with scores ranging from 0 (minimal complexity) to 21 (maximal complexity).

**Neighborhood disadvantage** was measured using the area deprivation index (ADI). This is calculated at the Census Block Group level using 17 census measures which capture information related to education, employment, income, poverty, and housing characteristics [40]. National ADI values were obtained from the Neighborhood Atlas website (https://www.neighborhoodatlas.medicine.wisc.edu/). National ADI values range from 1–100 and reflect national percentiles of neighborhood disadvantage with lower values reflect lower level of disadvantage within the nation. We categorized ADI into tertiles (Lowest deprivation, intermediate deprivation, and highest deprivation) based on the distribution of national ADI values across the wider HABS cohort [41].

**Past cognitive activity** was measured using the Cognitive Activities Scale [42]. Participants self-reported their frequency of engagement in cognitive activities at different points in their life at ages 6 (3 items), 12 (6 items), 18 (8 items), and 40 (8 items). Activities included visiting a library, reading newspapers, magazines, and books, and writing letters. Additional items were specific to age 6 e.g. being read to and telling a story. Frequency was assessed on a 5-point scale (1 = once a year or less; 2 = several times a year; 3 = several times a month; 4 = several times a week; 5 = everyday or almost everyday). Past cognitive activity was calculated as the average of the 25 items.

**Current cognitive activity** was measured using as the average of 11 items from the Cognitive Activities Scale. These items referred to the frequency of current engagement in cognitive activities.

A cross-sectional **CR residual** was computed by obtaining the standardized residual from a linear regression where baseline cognitive performance (PACC-5) was regressed on age, sex, neocortical Aβ, entorhinal tau, and adjusted hippocampal volume. Higher residual values reflect higher cognitive resilience.

We also examined two important risk factors as well as clinical progression. **APOE ε4 status** was assessed by direct genotyping of APOE from a blood sample. All ε4 haplotypes were considered as ε4 carriers by collapsing into a single category. **Cardiovascular disease risk** was assessed with the office-based Framingham Heart Study general cardiovascular disease (FHS-CVD) risk score [43] which provides a 10-year probability of future cardiovascular events (defined as coronary death, myocardial infarction, coronary insufficiency, angina, ischemic stroke, hemorrhagic stroke, transient ischemic attack, peripheral artery disease, and heart failure). This was calculated, from baseline data, as a weighted sum of age, sex, antihypertensive treatment (yes/no), systolic blood pressure (millimeters of mercury), body mass index, history of diabetes (yes/no), and current cigarette smoking status (yes/no) [44]. **Clinical progression** was assessed by calculating time to progression to the first of two consecutive nonzero CDR global scores (i.e. 0.5, 1, or 2) or to a final CDR global score of 0.5, 1, or 2.

### Statistical analyses

#### Latent class mixed models

Latent class mixture models are an extension of linear mixed models that can handle non-normally distributed longitudinal outcome variables and identify distinct subgroups of participants within a study population with trajectories that differ with respect to a latent variable [19]. We sought to select an appropriate model using LCMM that could identify distinct subgroups who differ based on our latent variable of interest, CR. We used the *lcmm* function (R package *lcmm*: https://cecileproust-lima.github.io/lcmm/) with cognition as the repeated measures outcome variable and time from cognitive baseline, age at cognitive baseline, biomarkers of AD pathology (neocortical Aβ and entorhinal tau), and neurodegeneration (adjusted hippocampal volume) as covariates. The covariates included in CR models vary across studies [3]. Some CR models have explicitly covaried for education [45–48] whereas many other studies did not adjust for education in their models [49–55]. We favored the latter approach, as we aimed to identify subgroups that differed by CR, and so we did not want to remove variance presumably related to CR from our model. Therefore, we did not include any CR-related factors as covariates in our model but instead investigated how these factors related to our model of CR in subsequent analyses.

We searched for a favored model across a combination of 4 different link functions (linear, beta, equidistant splines, and quantile spline link functions) – which normalize the repeated measures outcome variable – and different number of latent classes (2 – 7 classes). This resulted in a total of 24 candidate models. We first specified 1-class models for each link function to obtain initial values for the iterative estimation algorithm used in the subsequent analytic models (see eMethods for R code). To increase the likelihood of successful multi-class model convergence to the global maximum for each of the 24 candidate models, we implemented an automatic grid search with 30 iterations from 15 random departures from the initial values (see eMethods for R code).

#### Selection of favored latent class mixed model

As model fit statistics such as BIC values can decrease (becoming more favorable) as more model parameters are added, sole reliance on model fit statistics can lead to identification of an favored model that is overfit to the dataset and that may not identify clinically or theoretically reasonable trajectory classes [20, 21]. Therefore, we used a combination of model fit statistics, class discriminability, and theoretical considerations to identify our favored model [21].

**Fig. 1:**
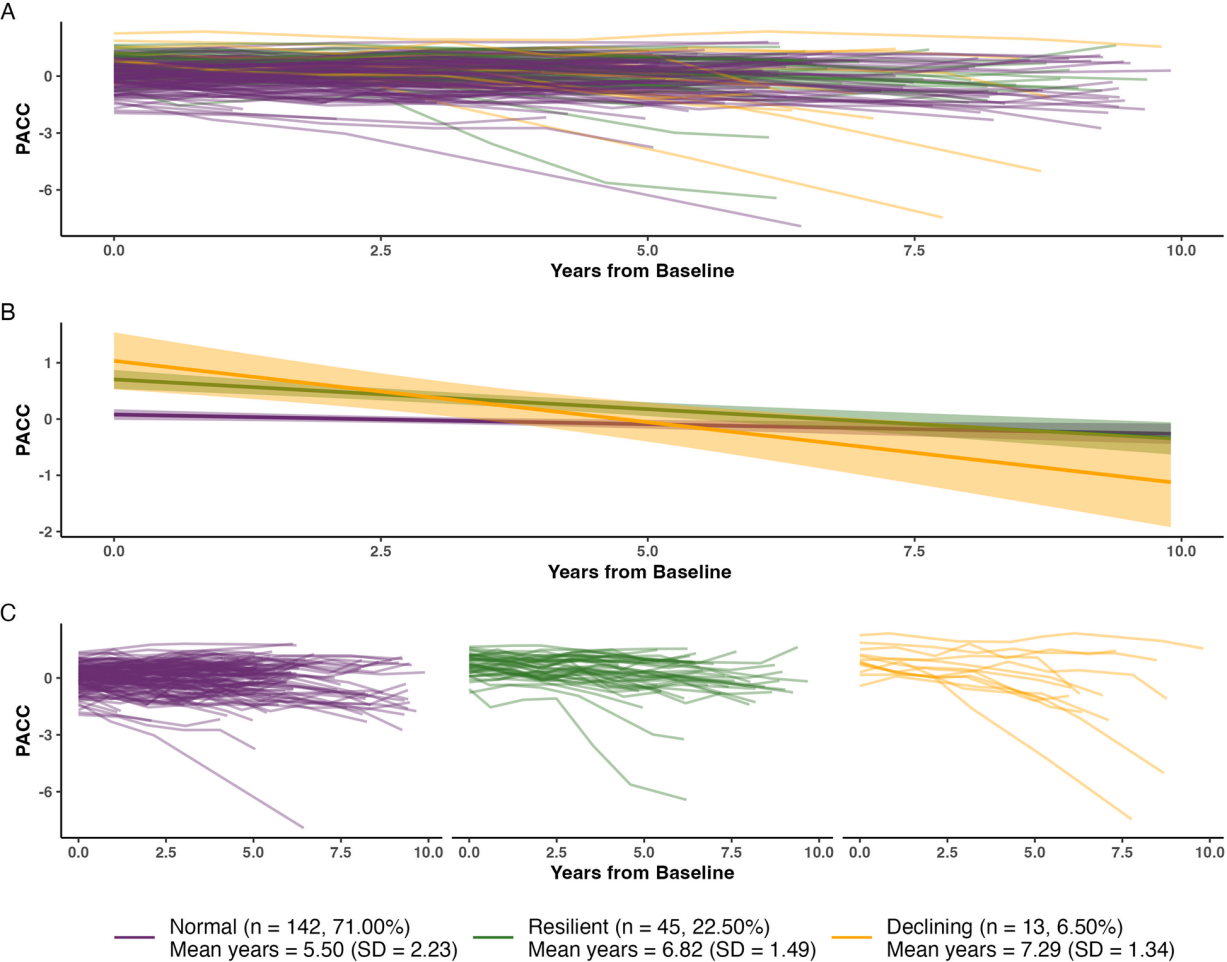
Separable latent subgroups of PACC-5 trajectories in HABS. **A** PACC-5 vs time from baseline (years) colored by latent subgroup. **B** Smoothed group-level trajectories for HABS colored by subgroup. **C** PACC-5 vs time from baseline (years) faceted and colored by subgroup

**Fig. 2:**
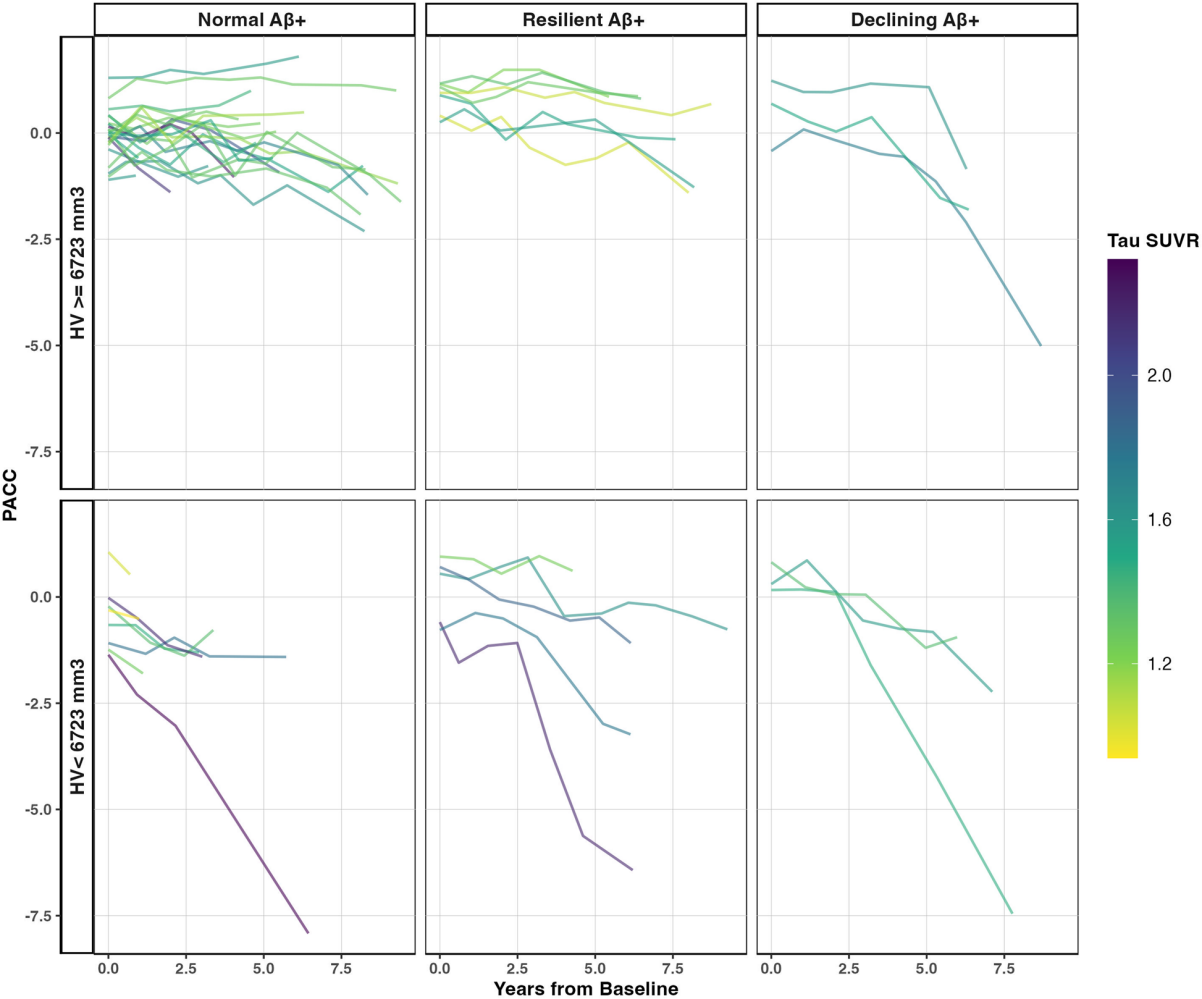
Examining PACC-5 trajectories in Aβ + participants suggests that the Resilient subgroup shows steep decline only in the presence of elevated Aβ, elevated tau and hippocampal atrophy. Aβ positivity was classified based on a threshold of > 1.185 DVR. Bottom panel shows trajectories of individuals who were positive for hippocampal atrophy (< 6,723mm). Top panel shows trajectories of individuals who were negative for hippocampal atrophy (> = 6,723mm). Aβ positivity and hippocampal atrophy were classified based on previously published thresholds < 6,723mm [57–59]

We first restricted the final set of candidate models to models that successfully converged and in which each identified trajectory class contained a meaningful proportion of the sample (i.e. at least 5% of the sample). We then identified a favored model by considering model fit statistics (sample size-adjusted BIC [SABIC] where lower values indicate better model fit), class discriminability statistics (relative entropy where values closer to 1 indicate less classification uncertainty [21, 56]) and theoretical reasoning (alignment with prior latent class analyses of cognitive trajectories and with post-mortem prevalence estimates of CR, and visual examination of group-level trajectories [see Fig. 1 and eFig. 2] and of individual trajectories with respect to Aβ, tau, and hippocampal volume [see Fig. 2]).

We then completed sensitivity analyses which consisted of comparing the selected model to the models with the same link function and number of subgroups (i.e. classes) but: 1) without covariates included (to confirm the baseline covariates provided useful information); 2) with inferior temporal tau SUVR in place of entorhinal tau SUVR; 3) with non-PVC tau SUVR data in place of PVC tau SUVR; and 4) only including individuals with at least 3 cognitive timepoints (to ensure that model selection was not unduly influenced by individuals with the minimum number of timepoints, i.e. 2 timepoints).

#### Comparison of CR-related factors and clinical progression across subgroups

We then compared CR-related factors across each latent trajectory subgroup in HABS using non-parametric Kruskal–Wallis tests with Dunn’s post hoc test for pairwise comparisons [60, 61]. Post hoc tests were corrected for multiple comparisons using the Hochberg method [62]. Pearson’s chi-squared (χ^2^) test was used to test for group differences in categorical variables. The time to clinical progression was compared across subgroups in a Kaplan–Meier analysis. Time to progression of the CDR global score (defined as an increase in the score from 0 and the event was classed as the first of two consecutive visits with a nonzero score or a final visit with a nonzero score). Data was censored at the time of last CDR assessment for individuals who did not show progression.

#### Application of favored latent class mixed model to ADNI data

Even with careful and considered model selection procedures, LCMMs may be highly dependent on idiosyncrasies specific to the cohort on which the model is derived [20]. As such, it has been recommended that papers using LCMMs adopt a predictive modeling/machine learning approach where the model selected in the original cohort is validated in an independent cohort [20]. To this end, we applied our selected model in the ADNI dataset. We conducted an LCMM with an automatic grid-search with the same model parameters as the selected model from HABS (i.e. using the same link function and number of classes). To ensure a stable solution, we repeated this procedure 10 times, and selected the most frequent model. We assessed whether this model successfully converged, identified meaningful trajectory subgroups and compared it to an unconditioned model (i.e. a model without covariates).

### Data availability statement

HABS data is available via submission of a data request here: https://habs.mgh.harvard.edu/researchers/request-data/. The HABS dataset and imaging protocols have been previously described [63]. ADNI data can be requested from LONI: https://adni.loni.usc.edu/.

## Results

### Identification of favored model

13 models successfully converged (see eTable 1) and 5 of these models were considered as candidate models as all classes contained a meaningful proportion of the sample [64] (> 5%; see Table 2). Among these candidate models (see eTable 2), the favored model comprised of a 3-class model using a splines link function (‘5-equi-splines’). The selected model displayed good model fit relative to all converged models based on SABIC, a recommended fit index for datasets with sample sizes smaller than 1,000 individuals [65]), acceptable discriminability (relative entropy > 0.5 [21]), and appeared to reveal meaningful and informative trajectory classes (see Fig. 1) with a sufficient N in each subgroup (largest subgroup = 71% of sample, next largest subgroup = 22.5% and smallest subgroup = 6.5%). Sensitivity analyses confirmed that this model was favored over alternative models (see eResults: Identification of favored model and sensitivity analyses for further information on model selection and sensitivity analyses).

**Table 2:** Characteristics of identified latent trajectories in HABS

**Characteristic**^1^	**Normal** *N* = 142^2^	**Resilient** *N* = 45^2^	**Declining** *N* = 13^2^	***p*-value**^3^
**Age**	71.61 (9.62)	71.76 (9.16)	75.38 (7.72)	0.3
**Sex**				0.7
F	82 (58%)	27 (60%)	9 (69%)	
M	60 (42%)	18 (40%)	4 (31%)	
**Race**				0.5
White	112 (81%)	39 (89%)	13 (100%)	
Black or African American	24 (17%)	5 (11%)	0 (0%)	
American Indian or Native Alaskan	2 (1.4%)	0 (0%)	0 (0%)	
American Indian or Native American/White	1 (0.7%)	0 (0%)	0 (0%)	
Unknown	3	1	0	
**Ethnicity**				0.6
Not Hispanic or Latino	140 (99%)	44 (98%)	13 (100%)	
Hispanic or Latino	2 (1.4%)	1 (2.2%)	0 (0%)	
**Neocortical Amyloid Burden**	1.17 (0.20)	1.16 (0.16)	1.27 (0.22)	0.2
**EC Tau Burden**	1.33 (0.28)	1.31 (0.25)	1.41 (0.38)	0.2
**Adj. HC Volume**	7,505.12 (807.85)	7,554.93 (912.03)	7,141.59 (824.10)	0.3
**APOE e4 Status** (*n* = 198)				0.4
e4-	105 (75%)	29 (64%)	9 (69%)	
e4 +	35 (25%)	16 (36%)	4 (31%)	
**PACC-5**	0.02 (0.65)	0.70 (0.55)	0.93 (0.71)	< 0.001
**CR Residual**	-0.34 (0.87)	0.71 (0.75)	1.21 (0.90)	< 0.001
**Years of Education**	15.96 (2.93)	16.91 (2.48)	17.54 (2.73)	0.026
**AMNART VIQ** (*n* = 199)	120.77 (8.94)	126.09 (5.38)	124.00 (6.39)	< 0.001
**Occupational Complexity** (*n* = 196)	10.05 (3.33)	10.25 (3.34)	10.62 (2.69)	0.6
**National ADI** (*n* = 195)				0.7
Lowest Tertile (1–7)	50 (36%)	17 (40%)	7 (58%)	
Intermediate Tertile (8–17)	51 (36%)	16 (37%)	3 (25%)	
Highest Tertile (18–85)	39 (28%)	10 (23%)	2 (17%)	
**Past Cognitive Activity** (*n* = 173)	2.97 (0.65)	2.83 (0.40)	2.44 (0.49)	0.008
**Current Cognitive Activity** (*n *= 178)	2.78 (0.49)	2.77 (0.39)	2.60 (0.44)	0.4
**CVD Risk** (*n* = 190)	26.76 (18.43)	26.05 (16.39)	27.62 (20.15)	> 0.9

^1^Data obtained within 1 year of analysis baseline except for the following variables, collected at full study baseline or prior to analysis baseline: APOE e4 Status, Years of Education, AMNART VIQ, Occupational Complexity, National ADI, Past Cognitive Activity, and CVD Risk

^2^Mean (SD); n (%) ^3^Kruskal-Wallis rank sum test; Pearson's Chi-squared test; Fisher's exact test

### Three separable cognitive trajectories relative to age, sex, and biomarkers of AD pathology and neurodegeneration.

The largest of the 3 subgroups identified in HABS (see Fig. 1) had a lower cognitive baseline level with a relatively stable cognitive trajectory given demographic and biomarker covariates (‘Normal’ subgroup, n = 142, 71% of sample). The next largest subgroup was labelled as ‘Resilient’ as they showed a higher baseline cognitive level and relatively stable trajectories given biomarker levels (‘Resilient’ subgroup, n = 45, 22.5%). The smallest subgroup had a higher baseline cognitive level with a steeper declining trajectory (‘Declining’ subgroup, n = 13, 6.5%). A linear mixed effects model confirmed that subgroups significantly differed on PACC-5 intercepts and slopes (see eFig. 4 and eTable 4). By definition, the subgroups did not differ on baseline age, sex, or baseline AD pathology or neurodegeneration (see Table 2). In a sensitivity analysis, PACC-5 scores at final observations were significantly lower in the Declining subgroup compared to the Normal and Resilient subgroups, suggesting that the Declining subgroup showed true decline rather than a regression to the mean effect [66] (see eTable 5).

### Subgroups did not differ on regional tau-PET SUVR

To further examine if the subgroups in HABS differed on baseline tau levels in other areas of the brain, a region-of-interest analysis was conducted where each tau SUVr (from 33 bilateral FreeSurfer regions) was regressed on subgroup in a linear regression (tau SUVr ~ Subgroup). After correction for multiple comparisons, the Resilient subgroup did not significantly differ from the Normal subgroup or the Declining subgroup on any regional tau SUVr values (see Table 3).

**Table 3:** Region-of-interest analysis for tau-PET SUVr according to latent class

ROI	Estimate	p-value	FDR p-value
bankssts	0.042	0.448	0.976
caudal anterior cingulate	-0.067	0.311	0.976
caudal middle frontal	-0.026	0.617	0.976
cuneus	0.089	0.102	0.976
frontal pole	0.089	0.277	0.976
fusiform	0.008	0.870	0.976
inferior parietal	0.023	0.615	0.976
inferior temporal	0.034	0.543	0.976
insula	-0.049	0.309	0.976
isthmus cingulate	-0.045	0.377	0.976
lateral occipital	0.094	0.156	0.976
lateral orbitofrontal	-0.013	0.813	0.976
lingual	0.044	0.330	0.976
medial orbitofrontal	-0.029	0.643	0.976
middle temporal	0.012	0.804	0.976
paracentral	-0.137	0.015	0.490
parahippocampal	0.029	0.608	0.976
pars opercularis	0.043	0.418	0.976
pars orbitalis	0.012	0.849	0.976
pars triangularis	0.036	0.507	0.976
pericalcarine	0.063	0.325	0.976
postcentral	-0.057	0.151	0.976
posterior cingulate	-0.003	0.946	0.976
precentral	0.010	0.786	0.976
precuneus	-0.045	0.384	0.976
rostral anterior cingulate	-0.024	0.694	0.976
rostral middle frontal	-0.001	0.980	0.980
superior frontal	-0.016	0.745	0.976
superior parietal	-0.006	0.907	0.976
superior temporal	0.004	0.934	0.976
supramarginal	0.023	0.595	0.976
temporal pole	0.004	0.943	0.976
transverse temporal	-0.032	0.682	0.976

*Note: FDR p-values from an FDR-corrected linear regression of tau-PET SUVr on latent class across 33 bilateral ROIs*

### Steep cognitive decline in Resilient subgroup requires Aβ positivity, elevated tau and hippocampal atrophy

As a visual examination of the face validity of the Resilient subgroup trajectory in HABS, Aβ positive subgroups of each latent classes were created by selecting only individuals with cortical Aβ above previously published thresholds of > 1.185 DVR [57–59] (see Fig. 2). Some individuals in the Resilient subgroup do eventually show steep cognitive decline, but these individuals have elevated Aβ, elevated tau, and reduced hippocampal volume at baseline and yet appear to show little decline over 2.5 years. Moreover, for these individuals, this steep decline occurs after the onset of clinical symptoms, in line with the inflection point that has been reported in individuals with high CR [67] (see eFig. 5).

### Differences in CR-related factors in the Resilient subgroup

**Fig. 3:**
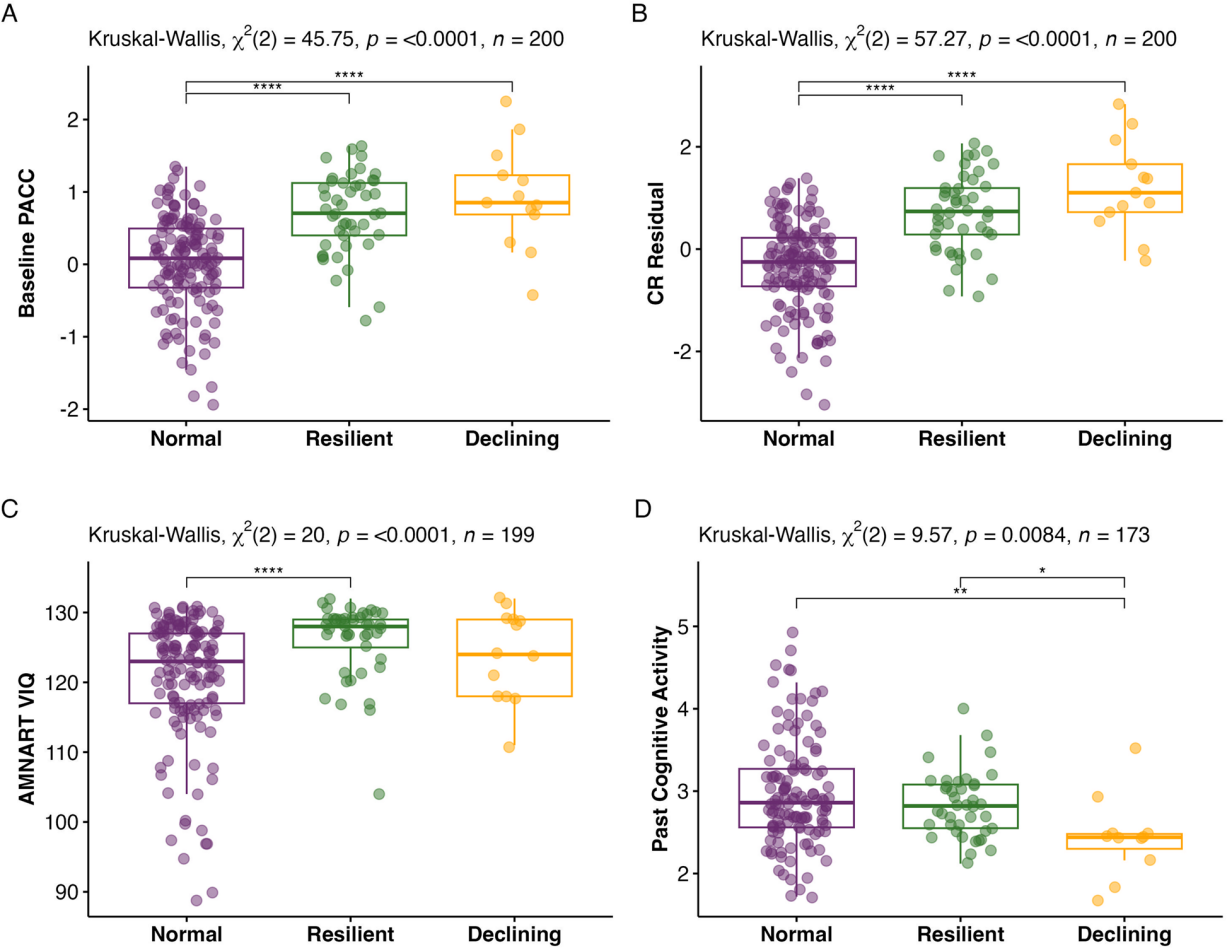
Pairwise comparisons of characteristics with significant differences across latent trajectory subgroups. **A** Baseline PACC-5. **B** CR residual. **C** AMNART VIQ. **D** Past Cognitive Activity

Both the Resilient and Declining subgroups had significantly higher baseline PACC-5 scores and CR residual values than the Normal subgroup (see Figs. 3A-B). The Resilient subgroup had significantly higher levels of verbal intelligence compared to the Normal subgroup (see Fig. 3C). The Resilient subgroup had numerically higher levels of verbal intelligence than the Declining subgroup, but this pairwise comparison was not statistically significant. Both the Resilient and Normal subgroups reported higher lifetime engagement in cognitive activities (Past Cognitive Activity) than the Declining subgroup (see Fig. 3D). Educational attainment was significantly different between the subgroups, but post-hoc pairwise comparisons did not discriminate between the groups (see eTable 6 for all post-hoc pairwise comparisons). No other differences were observed across subgroups on CR-related factors, e4 status, or CVD risk (see Table 2). A Kaplan–Meier survival analysis model revealed no differences in time to clinical progression (χ^2^ = 1.2, *p* = 0.56, see eFig. 6) across the 3 subgroups (16% progressed in the Normal subgroup, 24% in the Resilient subgroup, and 33% in the Declining subgroup).

### Application of selected LCMM to ADNI data

**Fig. 4:**
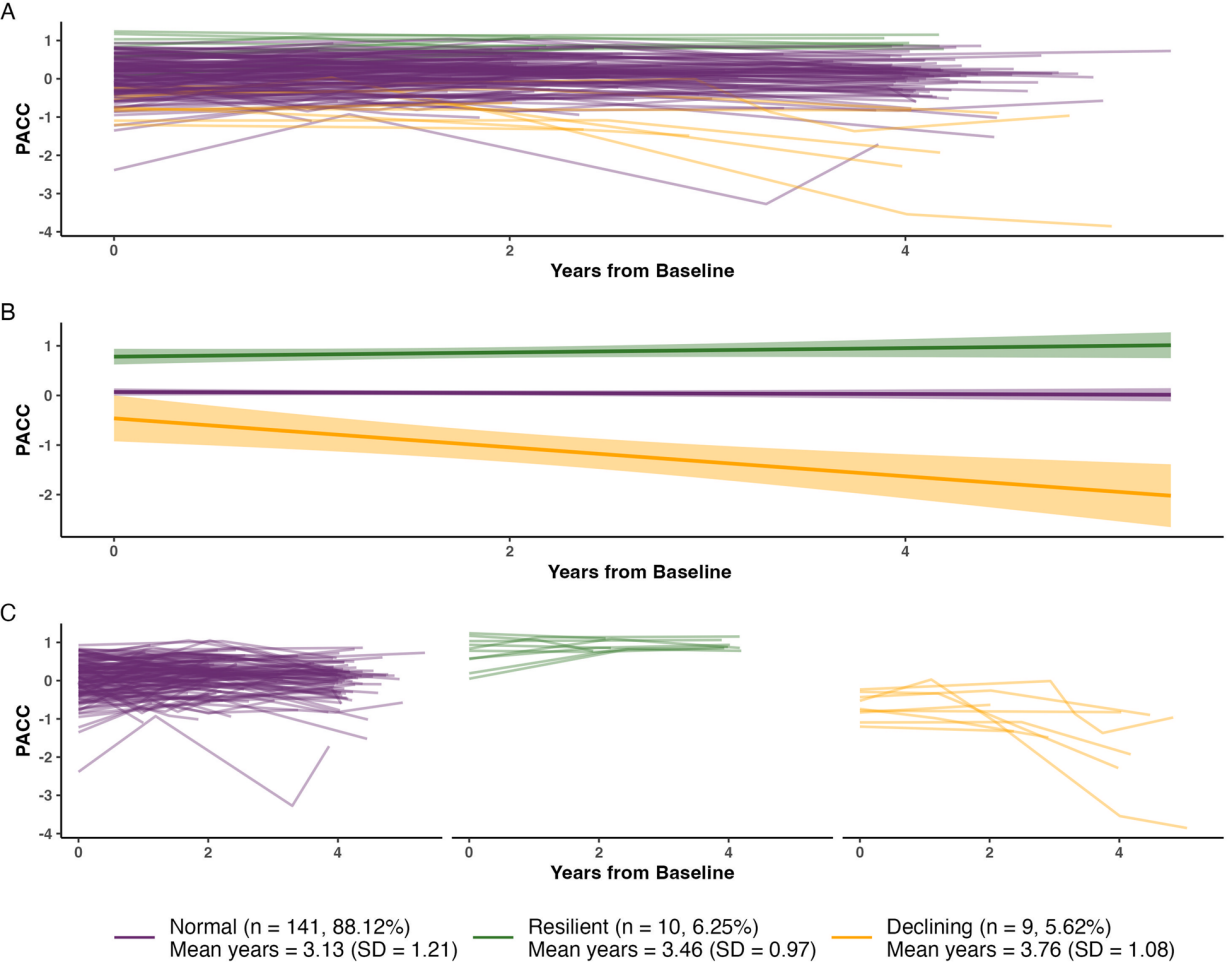
Separable subgroups of PACC-5 trajectories in ADNI. **A** PACC-5 vs time from baseline (years) colored by subgroup. **B** Smoothed group-level trajectories for HABS colored by subgroup. **C** PACC-5 vs time from baseline (years) faceted and colored by subgroup

Application of the selected model from HABS in the ADNI dataset identified similar subgroups (see Fig. 4). The model successfully converged, displayed good model fit (SABIC = 535.486) and discriminability (relative entropy = 0.847). This model showed better model fit and discriminability than an unconditioned model (SABIC = 557.035, relative entropy = 0.743, minimum class proportion = 1.88%). The largest subgroup had a lower cognitive baseline level and a stable cognitive trajectory (‘Normal’ subgroup, *n* = 141, 88.12% of sample). The next largest subgroup had a higher baseline cognitive level with a stable trajectory (‘Resilient’ subgroup, *n* = 10, 6.25%). The smallest subgroup had the lowest baseline cognitive level with a steeper declining trajectory (‘Declining’ subgroup, *n* = 9, 5.62%). A notable difference between the identified subgroup in HABS and ADNI was that the Declining subgroup in ADNI had a lower baseline level. The Resilient and Normal subgroups also remained on distinct group-level trajectories (i.e. confidence intervals did not overlap) in ADNI whereas in HABS, the confidence intervals of the Resilient and Normal subgroups begin to overlap at approximately 5.5 years after baseline. This occurred beyond the mean follow-up duration in ADNI (3.91 years) and the confidence intervals of the group-level trajectories of the HABS subgroups do not overlap when limited to this time period (see eFig. 7). As expected, subgroups did not differ on the model covariates, including baseline age, sex, and biomarkers of AD pathology and neurodegeneration (see eTable 7).

## Discussion

In a cohort of older adults from HABS, cognitively unimpaired at baseline, we identified 3 separable subgroups (Normal, Resilient, Declining) whose cognitive trajectories were statistically distinguishable after adjusting for baseline age, sex, neocortical Aβ, entorhinal tau, and adjusted hippocampal volume. The Resilient subgroup (22.5% of cohort) showed a high baseline cognitive level and a stable slope over a 10-year follow-up period. Nearly a quarter of the HABS participants were assigned to this cognitively resilient subgroup, in line with estimates of 20–38.8% from post-mortem studies, including the Adult Changes in Thought Study [8], the 90+ Study [7], and ROSMAP [9].

We found that the Resilient subgroup showed significantly higher premorbid verbal intelligence and levels of past cognitive activity. CR has been consistently associated with higher verbal intelligence [10, 38, 52, 68–70] and lifetime cognitive activity levels [10, 26, 52, 55]. Verbal intelligence may be a particularly important factor in CR as exposure to broader educational (i.e. including, but not restricted to, formal education in early life) and cognitive experiences over the lifespan, may be reflected in a better reading ability [71, 72]. Similarly, our findings suggest that regular engagement in cognitive activities may be critical in the development of CR over the lifespan [55]. This association may be specific to early and midlife periods, as the subgroups did not differ on current cognitive activity. A stronger association of early and mid-life experiences with CR, compared to late-life experiences, has been previously reported [55].

We observed numerically higher years of education in the Resilient subgroup compared to the Normal subgroup, although post-hoc comparisons were not statistically significant. This difference is not entirely surprising as, in contrast to verbal intelligence, the evidence relating educational attainment to CR is somewhat mixed, despite its widespread use as a proxy of CR [10, 11]. Education may be associated with cognitive decline in AD through other pathways than CR via its associations with general health or socioeconomic status, for example [55].

The Resilient subgroup did not show significantly higher occupational complexity nor did they display lower CVD risk. While previous findings have related increased occupational complexity to higher CR [73, 74], null associations have also been reported [10]. More nuanced consideration of occupational complexity, by quantifying specific job demands and skills, may better unravel the contribution of occupational complexity to CR [75]. While a composite CVD risk score was not associated with CR here, specific CVD risk factors, namely smoking and diabetes mellitus, have been associated with lower CR in other studies [76]. The contribution of CVD risk to CR may therefore be limited to specific CVD risk factors rather than overall CVD risk. Alternatively, we may have failed to detect an association with lower CVD risk as CVD risk was measured approximately 2.5 years before the measurement of cognitive and imaging data used to classify CR.

The Resilient subgroup demonstrated higher baseline values on the CR residual, which suggests that Resilient individuals possess higher cognitive reserve (i.e. referring to overall cognitive resources available [2]) and it may be this initially higher reserve that sets them apart (i.e., they have ‘more to lose’). However, sole reliance on cognitive level or measures of CR derived from cross-sectional data may not be a suitable approach for identifying individuals with CR [18] as the Declining subgroup also showed higher CR residual values at baseline, compared to the Normal subgroup. The faster subsequent decline, relative to baseline age, sex, AD pathology, and neurodegeneration, evident in the Declining subgroup highlights the importance of considering both level and slope of cognition when assessing CR. In line with this, using retrospective cognitive data from post-mortem cases, Wagner et al. compared different quantitative measures of CR and found that only a measure which considered both level and slope of cognition showed consistent associations with established CR factors [26].

In addition to adjusting for AD pathology in our model, we controlled for adjusted hippocampal volume to remove variance related to neurodegeneration downstream of AD pathology. This approach also removed variance related to brain reserve [4], which we consider sensible as we aimed to identify those who showed better-than-expected cognitive trajectories given adverse brain change and pathology [2]. This is in line with other approaches for investigating CR, which include testing interactions between CR-related variables and hippocampal volume on cognitive outcomes [10, 77, 78], or using a CR residual, where measures of cognitive performance are regressed on hippocampal volume [45, 54]. Our model effectively extends such approaches by also incorporating AD-specific biomarkers of Aβ and tau pathology. Given that hippocampal atrophy is not specific to AD pathology, and is associated with other factors such as TDP-43 [79] and vascular pathology [80], individuals identified as resilient in our model could potentially exhibit resilience beyond AD-specific pathology. Future work, incorporating biomarkers of other pathologies, could attempt to assess this.

Our model presents a novel alternative to the reliance on proxy variables or residual measures when characterizing CR and identifies a subgroup that appear to be cognitively resilient. We have provided R code in the supplementary materials so that other researchers can apply our model to investigate CR in their own dataset. Nonetheless, there were some limitations to our findings. The small size of the Declining group in HABS may have reduced statistical power to detect group-level differences in important variables, such as AD pathology, neurodegeneration, and clinical progression. A longer follow-up period would also improve the likelihood of detecting group-level differences in clinical progression. The varying follow-up periods for each individual, an inevitable consequence of analyzing data from ongoing observational studies, can lead to group differences in follow-up duration, as seen here where the Normal subgroup had shorter average follow-up durations than the Resilient and Declining subgroups. Future work could attempt to incorporate metrics such as Aβ chronicity to account for duration of exposure to elevated Aβ, which is associated with faster cognitive decline in cognitively unimpaired indiviudals [81]. As LCMMs may be strongly influenced by idiosyncrasies specific to the cohort on which the model is derived, it is recommended, although rarely applied in practice, to examine the model in an independent cohort [20]. We tested our model in the ADNI dataset and confirmed that the model successfully converged and provided a better fit to the data than an unconditioned model. In ADNI, we identified three somewhat similar trajectory subgroups. Unlike in HABS, however, these subgroups remained on distinct group-level trajectories. This was likely due to the lower baseline level of the Declining group and the shorter follow-up duration in ADNI as Resilient and Normal subgroup trajectories were distinct over the same time-period in HABS. A smaller Resilient subgroup (6.25% of analysis sample) was observed, which could be due, in part, to lower PACC-5 performance at the baseline relative to HABS, or a higher proportion of e4 + carriers and higher entorhinal tau burden in ADNI given that CR-related differences in cognition are diminished with increasing levels of pathology [82, 83]. ADNI participants also tended to be older (although this difference was not statistically significant) and these differences, along with differences in follow-up duration, amyloid-PET tracers, and time between cognitive and biomarker assessments, may limit the generalizability of our model, derived in HABS, to the ADNI cohort. Application of this model to other cohorts will be necessary to confirm these findings. More broadly, the HABS cohort are mostly highly educated, non-Hispanic White older adults who live in neighborhoods with low levels of deprivation. As such, the generalizability of these findings to more representative populations may be limited. For instance, in contrast to our findings, CR has been associated with lower neighborhood deprivation in cohorts with higher average levels of deprivation [41] and with higher educational attainment in cohorts with very low levels of education [84]. Finally, while we consider those in the Resilient subgroup to show CR to AD, we must acknowledge that many individuals in our sample had low levels of Aβ and tau at baseline and may not be on the AD continuum. Nonetheless, the Resilient subgroup, as a whole, displayed expected characteristics of a high CR group. Future work in large biomarker-confirmed preclinical AD cohorts, such as the A4 study [85], will increase our understanding of the nature of CR in preclinical AD.

In summary, we applied latent class mixture modeling to identify a subgroup with cognitively resilient trajectories based on longitudinal cognition conditioned on baseline age, sex, and imaging biomarkers of AD pathology and neurodegeneration. Our model identifies that up to 22.5% of older adults, cognitively unimpaired at baseline, display CR to AD pathophysiology and these individuals show higher verbal intelligence and higher levels of early to mid-life cognitive activities, supporting the theoretical validity of our approach. This proposed model provides a new useful tool that may be helpful to advance our understanding of CR to AD.

### Supplementary Information

Supplementary Material 1.

## Data Availability

HABS data is available via submission of a data request here: https://habs.mgh.harvard.edu/researchers/request-data/. The HABS dataset and imaging protocols have been previously described^60^. ADNI data can be requested from LONI: https://adni.loni.usc.edu/. R code is provided in the supplementary materials for application of latent class mixture models to other datasets.
