# Supplementary material for: Identifying longitudinal cognitive resilience from cross-sectional amyloid, tau, and neurodegeneration
Source: Alzheimers Res Ther. 2024 Jul 3;16:148. doi: 10.1186/s13195-024-01510-y (PMC11220971; doi:10.1186/s13195-024-01510-y)
Supplement: Supplementary file 1 — Supplementary Material 1. [file 13195_2024_1510_MOESM1_ESM.docx]

# eMethods

*R code to obtain initial values for 1-class models*

library(lcmm)

init_model <- lcmm(PACC ~ PACC_time + PACC_time*AB_FLR + PACC_time*TAU_EC + PACC_time*MRI_HV_Adj + PACC_time*PACC_age + PACC_time *SEX, random = ~ PACC_time, subject=’ID’, ng=1, idiag=FALSE, data=data, link=link_function)

Where:

- PACC_time = time from baseline PACC measurement
- PACC_age = age (years) at baseline PACC measurement
- link_function = one of ‘linear’, ‘beta’, ‘5-equi-splines’, ‘5-quant-splines’

If a 1-class model failed to successfully converge, we repeated the analysis by increasing the maximum number of iterations in the optimization algorithm from the default setting of 100 iterations (using argument *maxiter = 100*).

*R code to search across 24 candidate models*

# create empty list to store candidate models

models_list <- c()

# Loop through classes (from 2 to 7 classes) and run model

for (i in 2:7) {

# Loop through each link function and run for that class

for (j in 1:length(links)) {

curr_model <- gridsearch(rep = 30, maxiter = 15, minit = init_models[[j]], lcmm(

PACC ~ PACC_time + PACC_time*AB_FLR + PACC_time*TAU_EC + PACC_time*MRI_HV_Adj + PACC_time*PACC_age + PACC_time *SEX,

random = ~ PACC_time, mixture = ~ PACC_time, subject = 'ID',

ng = i, link = links[j], idiag = FALSE, data = data))

# store in models_list

models_list <- append(models_list, list(curr_model))

}

}

Where:

- init_models = list of initial models for each link function
- links = list of strings specifying link functions (linear’, ‘beta’, ‘5-equi-splines’, ‘5-quant-splines’)

*R code to run favored 3-class model*

# Get initial model for ‘5-equi-splines’ link function with 3 classes

init_model <- lcmm(PACC ~ PACC_time + PACC_time*AB_FLR + PACC_time*TAU_EC + PACC_time*MRI_HV_Adj + PACC_time*PACC_age + PACC_time *SEX, random = ~ PACC_time, subject=’ID’, ng=1, idiag=FALSE, data=data, link=’5-equi-splines’)

# Run favored model

model_3g <- gridsearch(rep = 30, maxiter = 15, minit = init_model, lcmm(

PACC ~ PACC_time + PACC_time*AB_FLR + PACC_time*TAU_EC + PACC_time*MRI_HV_Adj + PACC_time*PACC_age + PACC_time *SEX,

random = ~ PACC_time, mixture = ~ PACC_time, subject = 'ID',

ng = 3, link = ‘5-equi-splines’, idiag = FALSE, data = data))

# eResults

**eFigure 1.** Box plots comparing time between neuroimaging measurements and between baseline cognitive measurements and neuroimaging measurements. A = Weeks between tau-PET and Aβ-PET. B = Weeks between tau-PET and MRI. C = Weeks between Aβ-PET and MRI. D = Weeks between baseline PACC and tau-PET. E = Weeks between baseline PACC and Aβ-PET. F = Weeks between baseline PACC and MRI.

**eTable 1. Model fit and discrimination statistics for all assessed LCMMs ranked by SABIC.**

| **Model Description** | | **Model Statistics** | | | | | | **Class Size (% of sample)** | | | | | | | |
| --- | --- | --- | --- | --- | --- | --- | --- | --- | --- | --- | --- | --- | --- | --- | --- |
| **Link Function** | **Classes** | **Parameters** | **Converged** | **AIC** | **BIC** | **SABIC** | **Entropy** | **Class 1** | **Class 2** | **Class 3** | **Class 4** | **Class 5** | **Class 6** | **Class 7** | **Minimum** |
| **5-equi-splines** | **2** | **24** | **1** | **1,247.628** | **1,326.788** | **1,250.753** | **0.7392356** | **10.0** | **90.0** |  |  |  |  |  | **10.0** |
| **5-equi-splines** | **3** | **27** | **1** | **1,251.897** | **1,340.952** | **1,255.413** | **0.5172262** | **22.5** | **6.5** | **71.0** |  |  |  |  | **6.5** |
| **5-quant-splines** | **2** | **24** | **1** | **1,256.732** | **1,335.891** | **1,259.857** | **0.7150913** | **11.0** | **89.0** |  |  |  |  |  | **11.0** |
| 5-quant-splines | 3 | 27 | 2 | 1,257.310 | 1,346.365 | 1,260.826 | 0.7799251 | 0.5 | 86.0 | 13.5 |  |  |  |  | 0.5 |
| 5-equi-splines | 4 | 30 | 1 | 1,257.897 | 1,356.846 | 1,261.803 | 0.3263422 | 32.5 | 6.5 | 61.0 | 0.0 |  |  |  | 0.0 |
| **beta** | **2** | **21** | **1** | **1,261.743** | **1,331.007** | **1,264.477** | **0.7512708** | **10.0** | **90.0** |  |  |  |  |  | **10.0** |
| 5-equi-splines | 7 | 39 | 2 | 1,260.915 | 1,389.549 | 1,265.993 | 0.6079855 | 0.5 | 31.0 | 8.0 | 2.5 | 15.5 | 42.5 | 0 | 0.0 |
| 5-equi-splines | 6 | 36 | 2 | 1,261.993 | 1,380.732 | 1,266.681 | 0.3539315 | 8.0 | 13.5 | 1.5 | 48.0 | 0.0 | 29.0 |  | 0.0 |
| 5-equi-splines | 5 | 33 | 1 | 1,263.897 | 1,372.741 | 1,268.194 | 0.2468720 | 44.5 | 6.5 | 49.0 | 0.0 | 0.0 |  |  | 0.0 |
| **beta** | **3** | **24** | **1** | **1,266.225** | **1,345.385** | **1,269.350** | **0.5074096** | **16.0** | **76.5** | **7.5** |  |  |  |  | **7.5** |
| beta | 4 | 27 | 2 | 1,266.630 | 1,355.684 | 1,270.145 | 0.6443483 | 1.0 | 85.5 | 7.0 | 6.5 |  |  |  | 1.0 |
| 5-quant-splines | 4 | 30 | 2 | 1,267.064 | 1,366.013 | 1,270.970 | 0.3190999 | 35.5 | 57.5 | 0.0 | 7.0 |  |  |  | 0.0 |
| 5-quant-splines | 5 | 33 | 2 | 1,268.986 | 1,377.830 | 1,273.283 | 0.4212580 | 0.5 | 14.0 | 0.0 | 85.0 | 0.5 |  |  | 0.0 |
| beta | 7 | 36 | 1 | 1,271.213 | 1,389.952 | 1,275.901 | 0.6814533 | 6.5 | 5.5 | 30.0 | 2.5 | 48.0 | 0.5 | 7 | 0.5 |
| beta | 5 | 30 | 1 | 1,272.595 | 1,371.544 | 1,276.501 | 0.3917009 | 1.0 | 79.5 | 0.0 | 6.0 | 13.5 |  |  | 0.0 |
| beta | 6 | 33 | 2 | 1,278.593 | 1,387.438 | 1,282.890 | 0.2951957 | 1.0 | 0.0 | 21.5 | 0.0 | 71.5 | 6.0 |  | 0.0 |
| 5-quant-splines | 6 | 36 | 2 | 1,279.664 | 1,398.403 | 1,284.351 | 0.1729090 | 33.0 | 7.5 | 48.5 | 0.0 | 11.0 | 0.0 |  | 0.0 |
| 5-quant-splines | 7 | 39 | 2 | 1,286.842 | 1,415.477 | 1,291.921 | 0.1312036 | 0.0 | 19.0 | 0.0 | 0.0 | 81.0 | 0.0 | 0 | 0.0 |
| linear | 3 | 22 | 1 | 1,409.506 | 1,482.069 | 1,412.371 | 0.9645962 | 1.5 | 96.5 | 2.0 |  |  |  |  | 1.5 |
| linear | 4 | 25 | 1 | 1,411.494 | 1,493.952 | 1,414.749 | 0.8965037 | 1.5 | 93.5 | 1.5 | 3.5 |  |  |  | 1.5 |
| linear | 5 | 28 | 2 | 1,419.403 | 1,511.756 | 1,423.049 | 0.5464511 | 54.0 | 1.5 | 2.0 | 42.5 | 0.0 |  |  | 0.0 |
| linear | 6 | 31 | 1 | 1,421.039 | 1,523.287 | 1,425.075 | 0.6527859 | 1.0 | 60.0 | 1.5 | 32.0 | 1.0 | 4.5 |  | 1.0 |
| linear | 7 | 34 | 2 | 1,427.041 | 1,539.184 | 1,431.468 | 0.5730865 | 1.0 | 59.0 | 1.0 | 4.5 | 1.5 | 33.0 | 0 | 0.0 |
| linear | 2 | 19 | 1 | 1,432.206 | 1,494.874 | 1,434.680 | 0.9904841 | 98.5 | 1.5 |  |  |  |  |  | 1.5 |

*Note: Rows in bold denote candidate models as they contained a meaningful proportion of the sample (i.e. > 5% of sample per class).*

## Identification of favored model and sensitivity analyses

13 models successfully converged (see eTable 1). 5 of these models were considered as candidate models as they contained a meaningful proportion of the sample^1^ (> 5%). We ranked candidate models by SABIC, as SABIC is recommended in datasets with sample sizes smaller than 1,000 individuals^2^, and we considered relative entropy to reflect model discriminability between subgroups. The top-ranked model in terms of SABIC (and Entropy) was a 2-class model using a splines link function (‘5-equi-splines’). The next best model was a 3-class model using the same splines link function.

The 2-class model identified one small subgroup (‘Declining’ subgroup, 10% of sample) with a high baseline cognitive level and declining trajectory and one large subgroup (‘Normal’ subgroup, 90%) with a lower baseline cognitive level and a stable cognitive trajectory (see eFig. 2). The 3-class model identified smaller ‘Normal’ (71%) and ‘Declining’ subgroups (6.5%) with an additional medium-sized subgroup (‘Resilient’ subgroup, 22.5%) with a high baseline cognitive level and a stable cognitive trajectory.

We conducted sensitivity analyses to compare the two top-ranked LCMMs (based on SABIC) to alternative models with the same link function and number of classes but: 1) without covariates included (to confirm the baseline covariates provided useful information); 2) with inferior temporal tau SUVR in place of entorhinal tau SUVR (to confirm entorhinal tau was an appropriate measure of tau pathology in this sample); 3) with non-partial volume corrected entorhinal tau SUVR in place of partial volume corrected entorhinal tau SUVR (to confirm that partial volume corrected entorhinal tau SUVR was an appropriate measure of tau pathology in this sample); and 4) only including individuals with at least 3 cognitive timepoints (to ensure that model selection was not unduly influenced by individuals with the minimum number of timepoints, i.e. 2 timepoints).

For the alternative 2-class models, only the unconditioned model successfully converged. The favored 2-class model had better model fit (SABIC) but worse discriminability (Entropy) than the unconditioned model (see eTable 2). The alternative 3-class models successfully converged, with the exception of the non partial-volume corrected entorhinal tau model (see eTable 2).

The favored 3-class model had better model fit (lower SABIC) than an unconditioned model, a model using inferior temporal tau instead of entorhinal tau, and a model using non-partial volume corrected entorhinal tau instead of partial volume corrected entorhinal tau (see eTable 2). This model was also favored over an alternative model using a minimum of 3 timepoints per participant that had unacceptable discriminability (Entropy < 0.5), despite better model fit (lower SABIC). As such, the favored 3-class model was favored over all alternative models. The 3-class model had acceptable discriminability (Entropy > 0.5)^3^ and examination of residual plots for this model confirmed that the link function successfully normalized the longitudinal cognitive outcome variable (see eFig. 3).

We selected the 3-class model as our favored model based on theoretical reasoning. First, a systematic review of latent class analyses of cognitive trajectories in older adults identified that 3 to 4 classes are typically identified across studies^4^. Second, in line with the concept of CR, the Resilient subgroup displayed a higher-than-expected baseline cognitive level given pathology and degeneration and a stable trajectory (see Fig. 1). Third, when individuals in the Resilient subgroup showed steep cognitive decline, this occurred after approximately 2.5 years of relatively stable cognitive performance despite the presence of high baseline Aβ, tau and neurodegeneration (see Fig. 2). In contrast, in the Declining subgroup, steep cognitive decline occurred in the absence of high baseline tau or neurodegeneration and, in the Normal subgroup, steep decline occurred from baseline in those with high baseline burden (see Fig. 2). Finally, the proportion of individuals assigned to the Resilient subgroup by the favored model (22.5%) is in line with the previously reported proportions of individuals deemed to have exhibited CR in post-mortem studies (20-38.8%), including the Adult Changes in Thought Study^5^, the 90+ Study^6^, and ROSMAP^7^.

After selecting a 3-class model with a splines link function, with 5 equidistant nodes, we assessed whether the number of nodes was optimal for this model. We repeated the model, varying the number of nodes from 3-7 and identified 3 models that successfully converged (see eTable 3). The best-ranked model in terms of model fit (SABIC) and discriminability (Entropy) was the splines link function with 5 equidistant nodes.

**eTable 2. Sensitivity analyses for two candidate LCMMs (2-class and 3-class models with 5-equi-splines link function).**

| **Model Description** | | **Model Statistics** | | | | | | |
| --- | --- | --- | --- | --- | --- | --- | --- | --- |
| **Type** | **Classes** | **Converged** | **Parameters** | **AIC** | **BIC** | **SABIC** | **Entropy** | **% Per Class** |
| **Unconditioned** | 2 | Yes | 14 | 1,360.907 | 1,407.084 | 1,362.73 | 0.889 | 93.5, 6.5 |
| **Inferior temporal tau** | 2 | No | 24 | 1,277.011 | 1,356.170 | 1,280.136 | 0.830 | 93.5, 6.5 |
| **Non-PVC entorhinal tau** | 2 | No | 24 | 1,273.336 | 1,352.496 | 1,276.461 | 0.793 | 93.5, 6.5 |
| **3 timepoint minimum** | 2 | No | 24 | 1,222.273 | 1,300.201 | 1,224.179 | 0.734 | 93.5, 6.5 |
| **Unconditioned** | 3 | Yes | 17 | 1,356.878 | 1,412.949 | 1,359.091 | 0.918 | 92.5, 6.5, 1 |
| **Inferior temporal tau** | 3 | Yes | 27 | 1,265.354 | 1,354.409 | 1,268.87 | 0.506 | 59, 35, 6 |
| **Non-PVC entorhinal tau** | 3 | No | 27 | 1,279.373 | 1,368.428 | 1,282.889 | 0.870 | 93.5, 6.5, 0 |
| **3 timepoint minimum** | 3 | Yes | 27 | 1,217.764 | 1,305.433 | 1,219.909 | 0.308 | 88.4, 11.6, 0 |

*Note: Unconditioned = LCMM without any baseline covariates. Inferior temporal tau = LCMM using inferior temporal tau as a baseline covariate instead of entorhinal tau. Non-PVC entorhinal tau = LCMM using non-partial volume corrected entorhinal tau as a baseline covariate instead of partial volume corrected entorhinal tau. 3 timepoint minimum = LCMM using dataset restricted to participants with at least 3 cognitive timepoints (n = 190).*

**eTable 3. Comparison of different numbers of nodes in equidistant splines link function for 3-class LCMM.**

| **Model Description** | **Model Statistics** | | | | | | |
| --- | --- | --- | --- | --- | --- | --- | --- |
| **Nodes in Link Function** | **Converged** | **Parameters** | **AIC** | **BIC** | **SABIC** | **Entropy** | **% Per Class** |
| 3 | No | 25 | 1,254.602 | 1,337.060 | 1,257.858 | 0.508 | 73.5, 19, 7.5 |
| 4 | No | 26 | 1,253.589 | 1,339.345 | 1,256.975 | 0.517 | 61.5, 32, 6.5 |
| 5 | Yes | 27 | 1,251.897 | 1,340.952 | 1,255.413 | 0.517 | 71, 22.5, 6.5 |
| 6 | Yes | 28 | 1,257.196 | 1,349.549 | 1,260.842 | 0.291 | 88, 12, 0 |
| 7 | Yes | 29 | 1,253.772 | 1,349.423 | 1,257.548 | 0.510 | 70.5, 23, 6.5 |

*
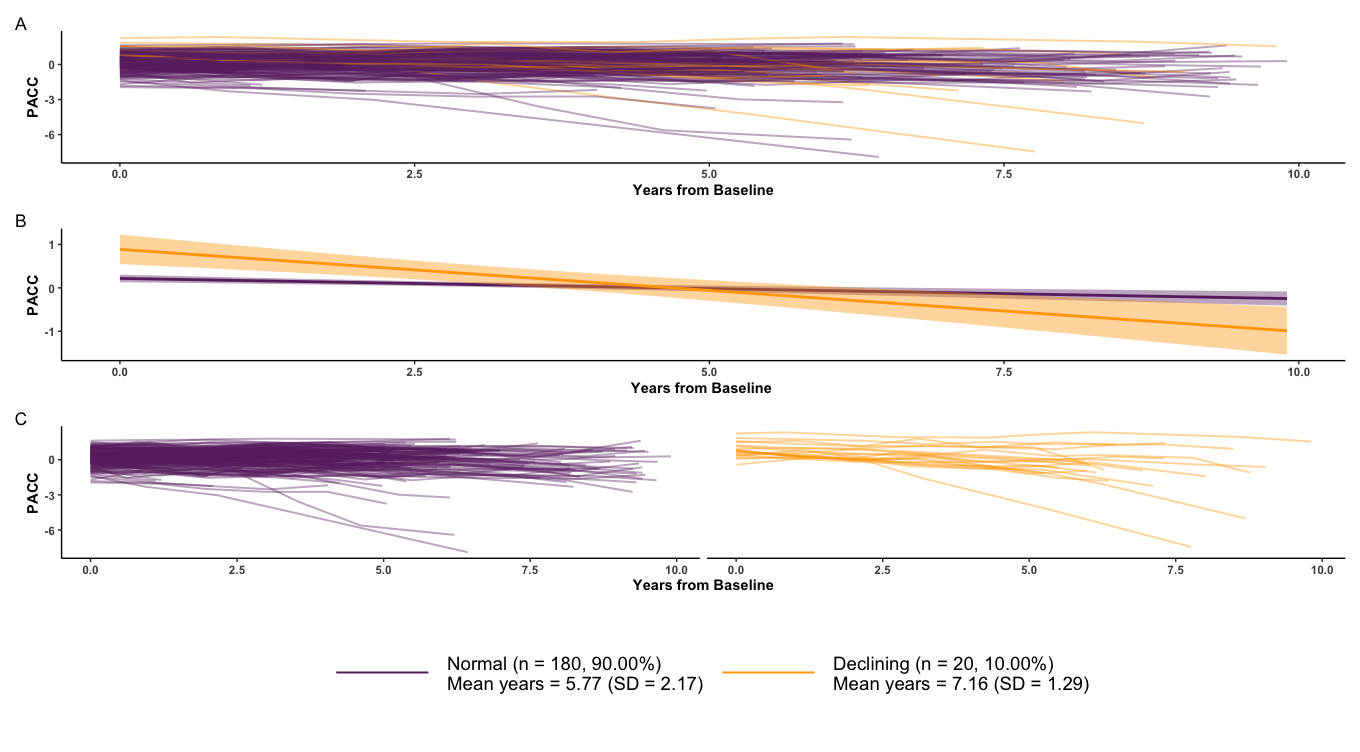
*

**eFigure 2. Latent class trajectories in HABS identified by 2-class LCMM with equidistant splines link function.** A = PACC vs time from baseline (years) colored by latent class. B = Smoothed group-level trajectories for HABS coloured by class. C = PACC vs time from baseline (years) faceted and coloured by class.

**
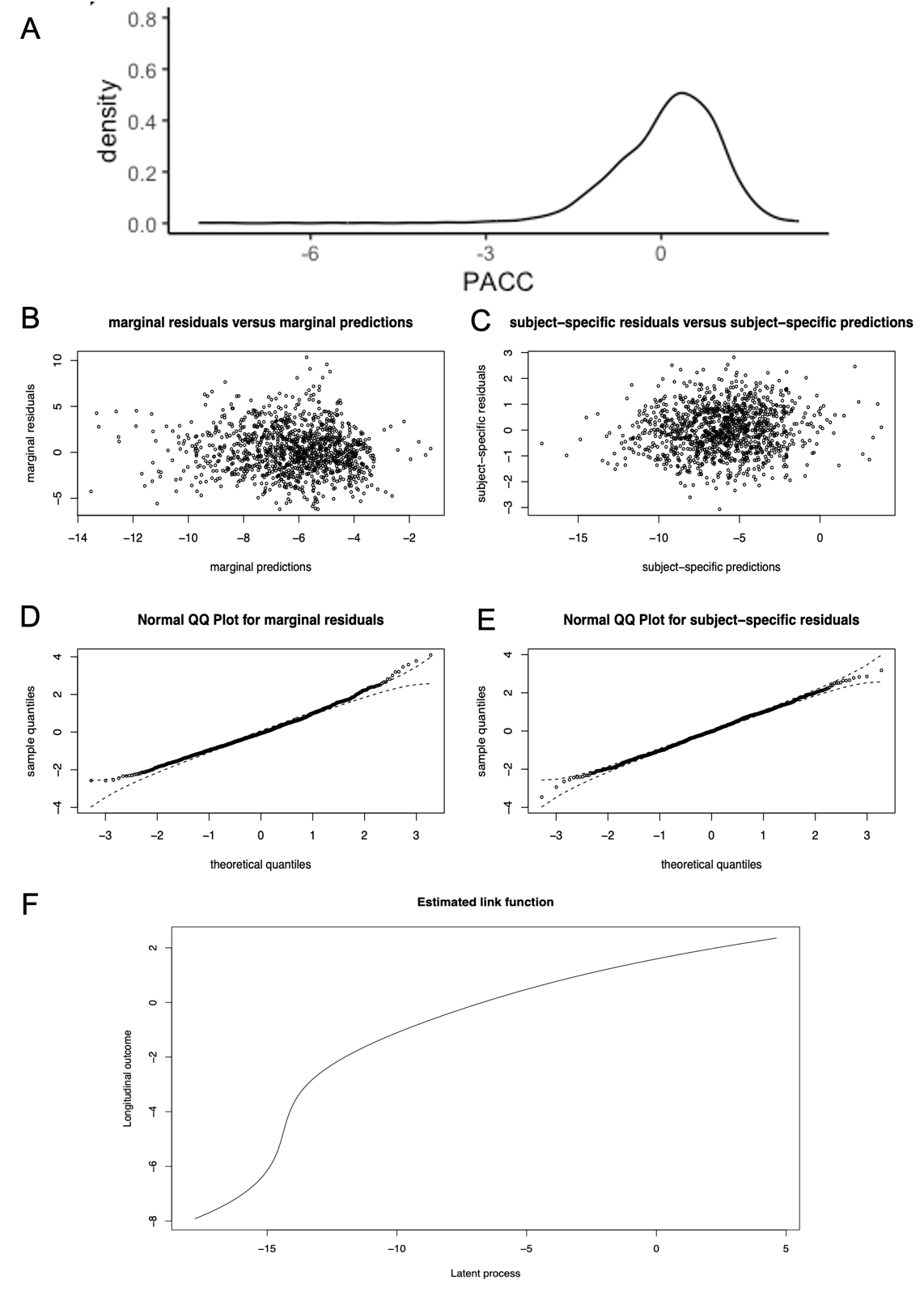
**

**eFigure 3. Model diagnostic plots for favored LCMM.** A = Kernel density estimate plots of the raw PACC distribution in HABS showing a left-skewed distribution. B = Marginal residuals versus marginal predicted values plot. BC = Subject-specific residuals versus subject-specific predicted values plot. D = Q-Q plot of the marginal residuals. ED = Q-Q plot of the subject-specific residuals. F = Estimated link function obtained in the LCMM.

**eTable 4. Linear mixed effects model of PACC5 over time on subgroup from LCMM.**

|  | **Estimate** | **CI** | **p** |
| --- | --- | --- | --- |
| Intercept | 0.735 | 0.568 – 0.902 | < 0.001 |
| Time from baseline | -0.116 | -0.161 – -0.072 | < 0.001 |
| Subgroup [Normal] | -0.673 | -0.866 – -0.480 | < 0.001 |
| Subgroup [Declining] | 0.448 | 0.093 – 0.802 | 0.014 |
| Time from baseline x Subgroup [Normal] | 0.064 | 0.012 – 0.115 | 0.016 |
| Time from baseline x Subgroup [Declining] | -0.191 | -0.284 - -0.098 | < 0.001 |

*Note: PACC-5 as outcome in a linear mixed effects model including time from PACC5 baseline*subgroup with random intercepts and random slopes.*

**eTable 5. Comparison of PACC5 at final observation in Declining subgroup compared to Normal and Resilient subgroups.**

|  | **β** | **CI** | **p** |
| --- | --- | --- | --- |
| Intercept |  |  | .0142 |
| Time from baseline | -0.027 | -0.12 – 0.07 | 0.713 |
| Subgroup [Normal] | 0.379 | -0.411 – 1.17 | 0.005 |
| Subgroup [Resilient] | 0.397 | -0.443 – 1.24 | 0.002 |

*Note: Linear regression of PACC-5 at final observation on subgroup adjusting for follow-up duration (time from baseline). Declining class set as the reference level in subgroup.*

*
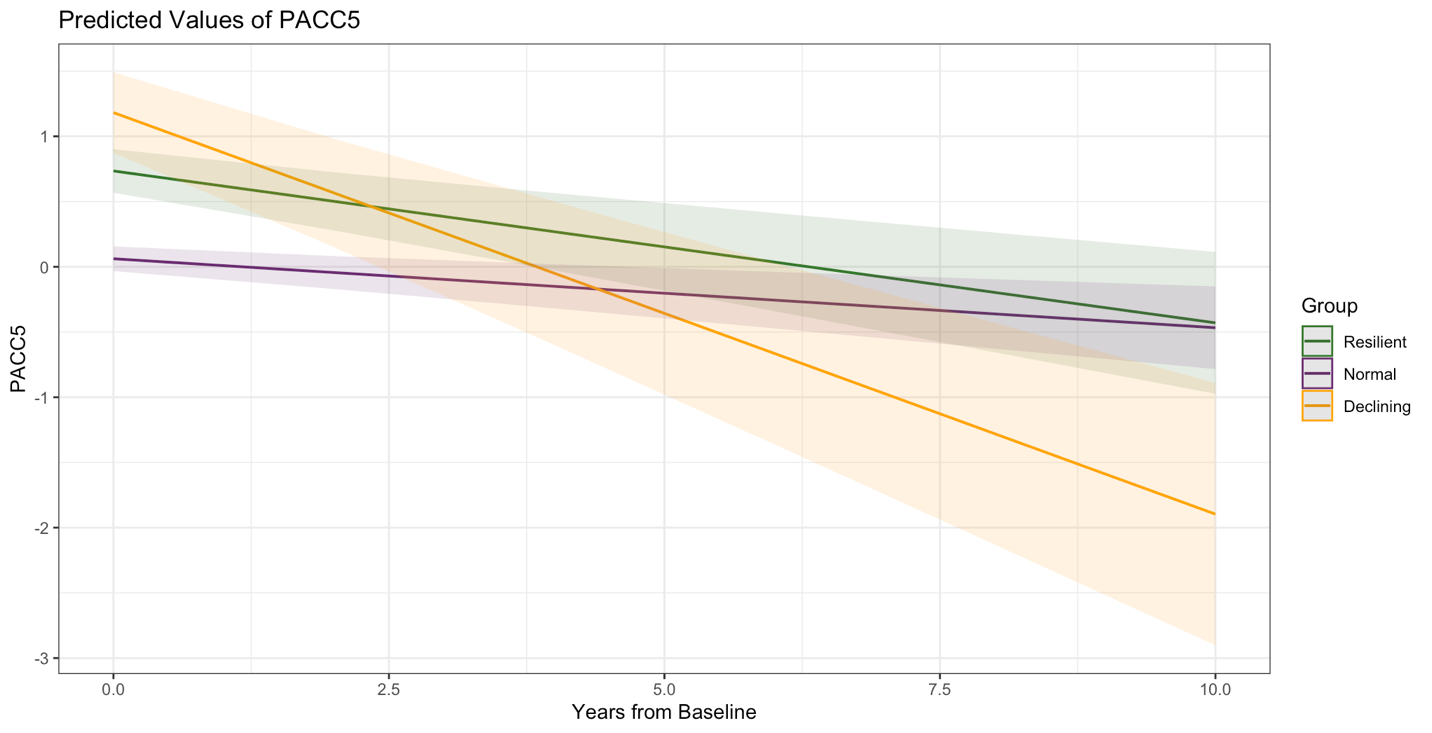
*

**eFigure 4. Subgroups show significantly different PACC5 intercepts and slopes.** The Declining subgroup show the highest PACC5 level with the steepest decline. The Resilient subgroup display a high PACC5 level with faster, but delayed, decline in comparison to the Normal subgroup.


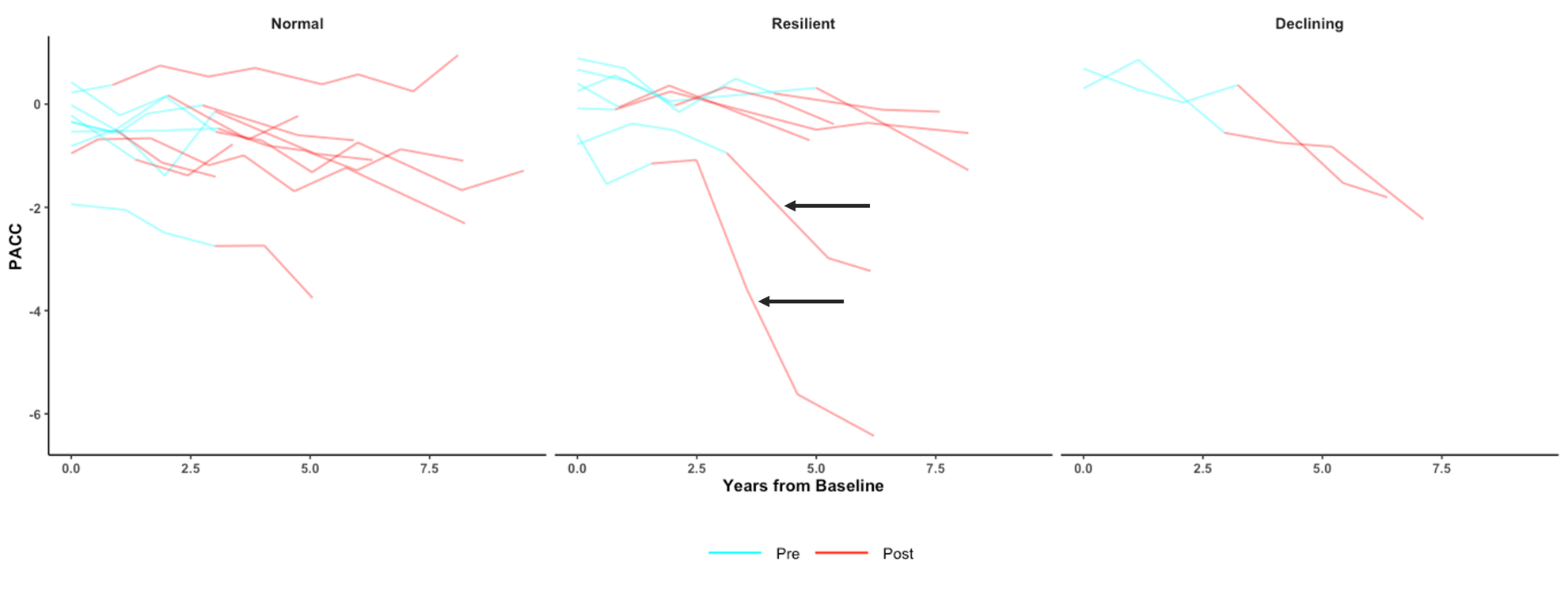


**eFigure 5. Steeper decline after clinical progression is evident in some individuals in the Resilient class.**Black arrows point to examples of trajectories in the Resilient class that show accelerated decline after the onset of clinical symptoms (i.e. nonzero CDR score) with cyan lines showing pre-symptom onset and red lines showing post-symptom onset. These two individuals had elevated Aβ, elevated tau and hippocampal atrophy at analysis baseline yet maintained normal cognitive function for at least 1.5 years before declining to the range of mild cognitive impairment, as indicated by CDR > 0, whereafter they displayed accelerated decline.

**eTable 6. Post-hoc tests for pairwise comparisons of baseline characteristics across identified trajectory classes.**

| **Characteristic** |  | **Group 1** | **Group 2** | **Kruskal-Wallis χ^2^** | **Corrected**  **p-value** |
| --- | --- | --- | --- | --- | --- |
| **PACC** |  | Normal | Resilient | 5.87 | <.001 |
|  |  | Normal | Declining | 4.16 | <.001 |
|  |  | Resilient | Declining | .642 | .521 |
| **CR Residual** |  | Normal | Resilient | 6.36 | <.001 |
|  |  | Normal | Declining | 4.97 | <.001 |
|  |  | Resilient | Declining | 1.12 | .264 |
| **Years of Education** |  | Normal | Resilient | 1.88 | .12 |
|  |  | Normal | Declining | 2.48 | .088 |
|  |  | Resilient | Declining | .982 | .326 |
| **AMNART VIQ** (n = 199) |  | Normal | Resilient | 4.40 | <.001 |
|  |  | Normal | Declining | 1.41 | .27 |
|  |  | Resilient | Declining | -1.1 | .27 |
| **Past Cognitive Activity** (n = 173) |  | Normal | Resilient | -.879 | .379 |
|  |  | Normal | Declining | -3.06 | .007 |
|  |  | Resilient | Declining | -.2.36 | .037 |

**
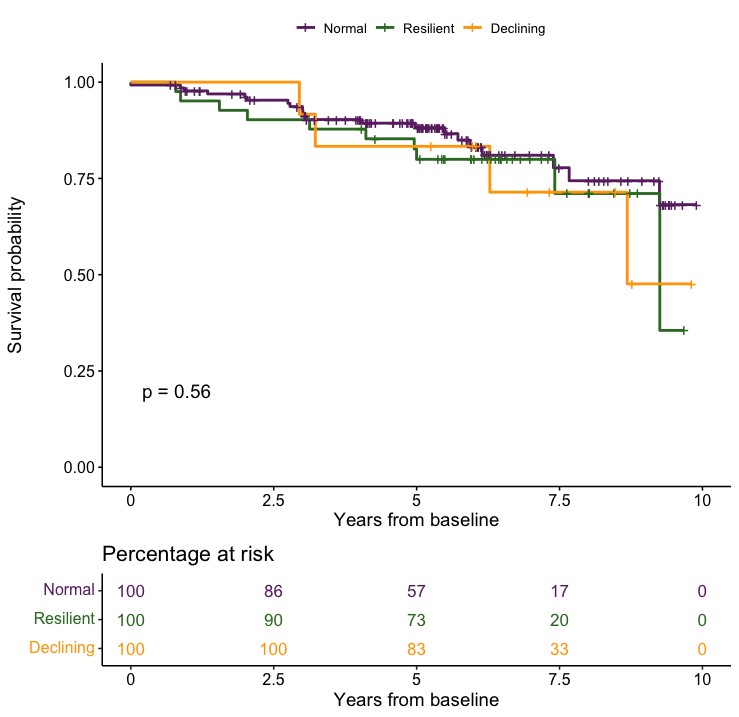
**

**eFigure 6. Time to clinical progression across trajectory classes in HABS.** Kaplan-Meier survival curves showing progression to CDR scores > 0 with the event defined as first of two consecutive visits or a final visit with a nonzero CDR score.

**eTable 7. Characteristics of identified latent trajectories in ADNI**

| **Baseline Characteristic** | **Normal**  N = 141^1^ | **Resilient**  N = 10^1^ | **Declining**  N = 9^1^ | **p-value**^2^ |
| --- | --- | --- | --- | --- |
| **Age** | 74.17 (7.5) | 72.73 (9.26) | 70.98 (7.35) | 0.4 |
| **Sex** |  |  |  | 0.6 |
| F | 83 (59%) | 6 (60%) | 7 (78%) |  |
| M | 58 (41%) | 4 (40%) | 2 (22%) |  |
| **Neocortical Amyloid Burden** | 1.15 (0.19) | 1.38 (0.14) | 1.48 (0.12) | 0.4 |
| **EC Tau Burden** | 1.72 (0.3) | 1.48 (0.35) | 1.54 (0.13) | 0.4 |
| **Adj. HC Volume** | 7,444.03 (830.99) | 7,158.16 (876.99) | 7,472.64 (622.89) | 0.6 |
| **APOE e4 Status** (n = 151) |  |  |  | 0.7 |
| e4+ | 54 (41%) | 4 (40%) | 2 (25%) |  |
| e4- | 79 (59%) | 6 (60%) | 6 (75%) |  |
| **PACC** | 0.05 (0.5) | 0.74 (0.4) | -0.68 (0.34) | <0.001 |

^1^Mean (SD); n (%) ^2^Kruskal-Wallis rank sum test; Fisher's exact test.


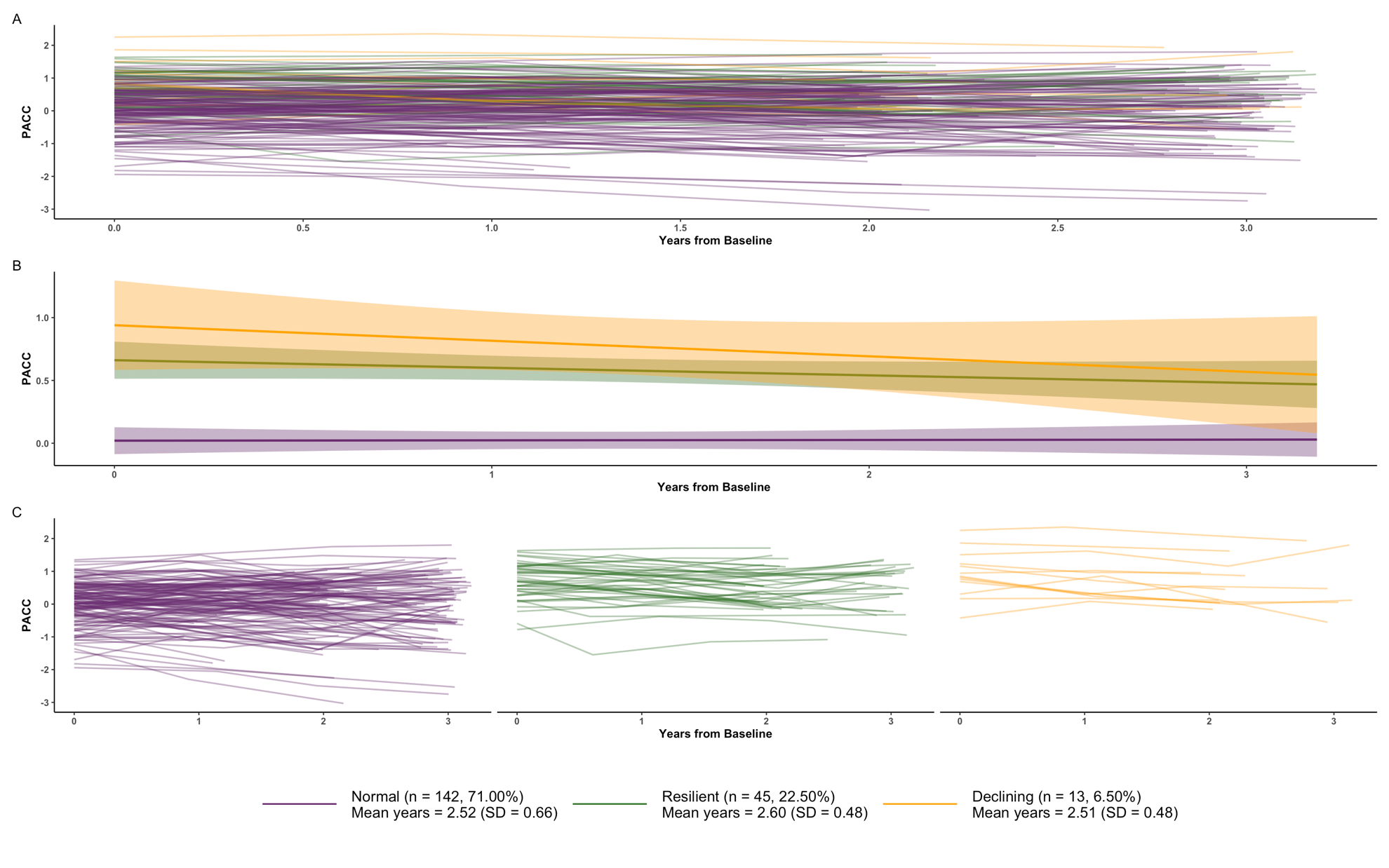
 **eFigure 7. PACC-5 trajectories in HABS restricted to the mean follow-up period in ADNI.** A = PACC-5 vs time from baseline (years) colored by subgroup. B = Smoothed group-level trajectories for HABS colored by subgroup. C = PACC-5 vs time from baseline (years) faceted and colored by subgroup.

1. Pietrzak RH, Lim YY, Ames D, et al. Trajectories of memory decline in preclinical Alzheimer’s disease: results from the Australian Imaging, Biomarkers and Lifestyle Flagship Study of Ageing. *Neurobiology of Aging*. 2015;36(3):1231-1238. doi:10.1016/j.neurobiolaging.2014.12.015

2. Chen Q, Luo W, Palardy GJ, Glaman R, McEnturff A. The Efficacy of Common Fit Indices for Enumerating Classes in Growth Mixture Models When Nested Data Structure Is Ignored: A Monte Carlo Study. *SAGE Open*. 2017;7(1):2158244017700459. doi:10.1177/2158244017700459

3. Lennon H, Kelly S, Sperrin M, et al. Framework to construct and interpret latent class trajectory modelling. *BMJ Open*. 2018;8(7):e020683. doi:10.1136/bmjopen-2017-020683

4. Wu Z, Phyo AZZ, Al-harbi T, Woods RL, Ryan J. Distinct Cognitive Trajectories in Late Life and Associated Predictors and Outcomes: A Systematic Review. *Journal of Alzheimer’s Disease Reports*. 2020;4(1):459-478. doi:10.3233/ADR-200232

5. Aiello Bowles EJ, Crane PK, Walker RL, et al. Cognitive Resilience to Alzheimer’s Disease Pathology in the Human Brain. *Journal of Alzheimer’s Disease*. 2019;68(3):1071-1083. doi:10.3233/JAD-180942

6. Robinson JL, Corrada MM, Kovacs GG, et al. Non-Alzheimer’s contributions to dementia and cognitive resilience in The 90+ Study. *Acta Neuropathol*. 2018;136(3):377-388. doi:10.1007/s00401-018-1872-5

7. Schneider JA, Aggarwal NT, Barnes L, Boyle P, Bennett DA. The Neuropathology of Older Persons with and Without Dementia from Community versus Clinic Cohorts. *Journal of Alzheimer’s Disease*. 2009;18(3):691-701. doi:10.3233/JAD-2009-1227
